# Developing a knowledge transfer and exchange strategy for a clinical trials unit

**DOI:** 10.1186/s13063-024-08681-x

**Published:** 2025-01-03

**Authors:** Annabelle South, Berta Terré Torras, Hannah Rush, Anna Goodman, Sharon Love

**Affiliations:** MRC Clinical Trials Unit at UCL, Institute of Clinical Trials and Methodology, UCL, 90 High Holborn, London, WC1V 6LJ UK

**Keywords:** Impact, Knowledge transfer and exchange, Strategy, Communication, Clinical trials

## Abstract

**Need for a strategic approach to knowledge transfer and exchange:**

Late-phase clinical trials and systematic reviews find results that have the potential to improve health outcomes for people. However, there are often delays in these results influencing clinical practice. We developed a knowledge transfer and exchange strategy to support research teams, aiming to identify activities along the research process to maximise and accelerate the research impact.

**Our knowledge transfer and exchange strategy:**

The strategy has five strands of activity across the life-course of our research:

1. Partnerships with external stakeholders (including patient and public involvement, charities, policymakers, healthcare professionals, professional bodies, regulators and industry)

2. Communication (including the development of research impact strategies and use of communication tools and channels)

3. Maximising the scientific value of our studies (including open access, data and sample sharing, and incorporating multi-disciplinary components within studies)

4. Strengthening capacity (including building internal and partner capacity to communicate effectively, and strengthening the capacity of external stakeholders to understand and apply our research).

5. Learning and sharing (evaluating the impact of research, sharing lessons learnt internally and externally)

The strategy has helped trial teams think systematically about impact and was easy to use.

**Conclusions:**

Our strategy helps researchers systematically identify activities which may improve the usefulness and uptake of their study results. While developed in a single trials unit, we think it may be of use to others.

**Supplementary Information:**

The online version contains supplementary material available at 10.1186/s13063-024-08681-x.

## Need for a strategic approach to knowledge transfer and exchange

In recent decades there has been increasing interest in the impact of research. Late phase clinical trials and systematic reviews of trials may find results that have the potential to improve health outcomes for people. However, there are often delays in the results influencing clinical practice. Previous research has found that it can take almost two decades, on average, for research results to go from discovery to practical application [1, 2]. These delays in implementing evidence-based approaches have serious implications for patients and the health care system. The most obvious effect is that, due to this delay, many patients and service users miss out on the benefits of evidence-based care [1–3]. These delays are not inevitable; for example, during the COVID-19 pandemic guidelines incorporating the latest evidence from trials and meta-analyses were developed at pace, and practice changed rapidly in response to emerging evidence [4].

Against this backdrop, the concept of knowledge transfer and exchange has developed, which seeks to encourage the movement of research knowledge into action [5]. Originally developed by the Canadian Institute of Health Research, many research funders now encourage grant applicants to think about how their research will be translated into action from this early stage of the development of ideas. This is of particular interest to public and charitable research funders, who want to be able to demonstrate to tax payers and donors that their investment in research has resulted in changes in policy and practice. Having a knowledge transfer and exchange strategy is a requirement of the Medical Research Council for University Units it funds, which includes our department. Part of the vision of our department is delivering a swifter and more effective translation of scientific research into patient benefits. Many models and frameworks to understand the knowledge to practice process exist [6–17], but these may be hard for busy clinical trialists to translate into practical actions. We therefore sought to develop a knowledge transfer and exchange strategy for our clinical trials unit, to support research teams to think through the actions they can take at different stages of their research to maximise and accelerate the impact of that research on policy and practice. This letter describes the strategy we developed, and how it was developed.

## Our context

The Medical Research Council Clinical Trials Unit at UCL (MRCCTU at UCL) is a large clinical trials unit carrying out mostly late-phase trials in the areas of infectious diseases, cancer and neurodegenerative diseases. We work in both high and low- and middle-income settings. Our aim is to deliver a swifter and more effective translation of our trial and meta-analysis results into health benefits. Effective knowledge transfer and exchange is essential to achieving this. We have a small team of research communications professionals who support the knowledge transfer and exchange activities of the unit.

## Development of the strategy

The first step in developing the strategy occurred at a senior staff away day, where attendees were asked to list the activities they did as part of their studies to encourage knowledge transfer and exchange. These activities were grouped into 5 ‘strands’, described in Table 1.

- Partnerships
- Communication
- Maximising the scientific value of our studies
- Strengthening capacity
- Learning and sharing

**Table 1:** Description of the strands of our knowledge transfer and exchange strategy

Strand	Description
Partnerships with external stakeholders	Including collaborators involved in implementing our research; patient and public involvement, and stakeholder engagement activities
Communication	Activities to communicate about our research to various audiences, throughout the study process
Maximising the scientific value of studies	Actions to ensure our studies generate the range of evidence needed by stakeholders (such as including multi-disciplinary sub-studies) and that evidence is accessible to stakeholders (such as through open access publications and data sharing)
Strengthening capacity	Including efforts to build the capacity of our staff and partners around knowledge transfer and exchange, and to build the capacity of stakeholders to understand and apply the results of our studies
Learning and sharing	Evaluating the impact of our studies and knowledge transfer and exchange work to inform future studies; sharing our learning internally and externally, and seconding people to and from other organisations, so we can learn and share our knowledge with them

We then formed a Knowledge Transfer and Exchange Working Group, made up of representatives from the Infections Cancer, and Methodology Research Themes together with members of the Communications Team. This group was tasked with developing the Knowledge Transfer and Exchange strategy for the unit. The group met approximately monthly throughout 2022. The group decided the strategy needed to cover activities that happen at the unit-level and those that happen at the study-level. It was agreed that there were substantial differences between the sorts of study-level activities appropriate for clinical trials, observational studies and meta-analyses, and those relevant for methodological research into the design, conduct and analysis of clinical trials and meta-analyses. A sub-group was formed to focus on developing a version that was relevant to methodological studies. This letter shares the strategy developed for clinical trials, observational studies, meta-analyses and other studies where primary data are being collected.

Activities were included in the strategy if they have been used in at least some of our studies. Those that were mandatory in order to comply with department or funder policies (such as open access publication, and patient and public involvement) were categorised as essential. Those that are likely to be useful and appropriate for most of our studies were highly recommended, while those which may only be relevant in some contexts (but useful in those situations) were categorised as for consideration. We excluded activities that, although known to be effective at promoting research impact, were unlikely to be feasible for our studies, such as academic detailing (outreach) interventions [18].

**Fig. 1:**
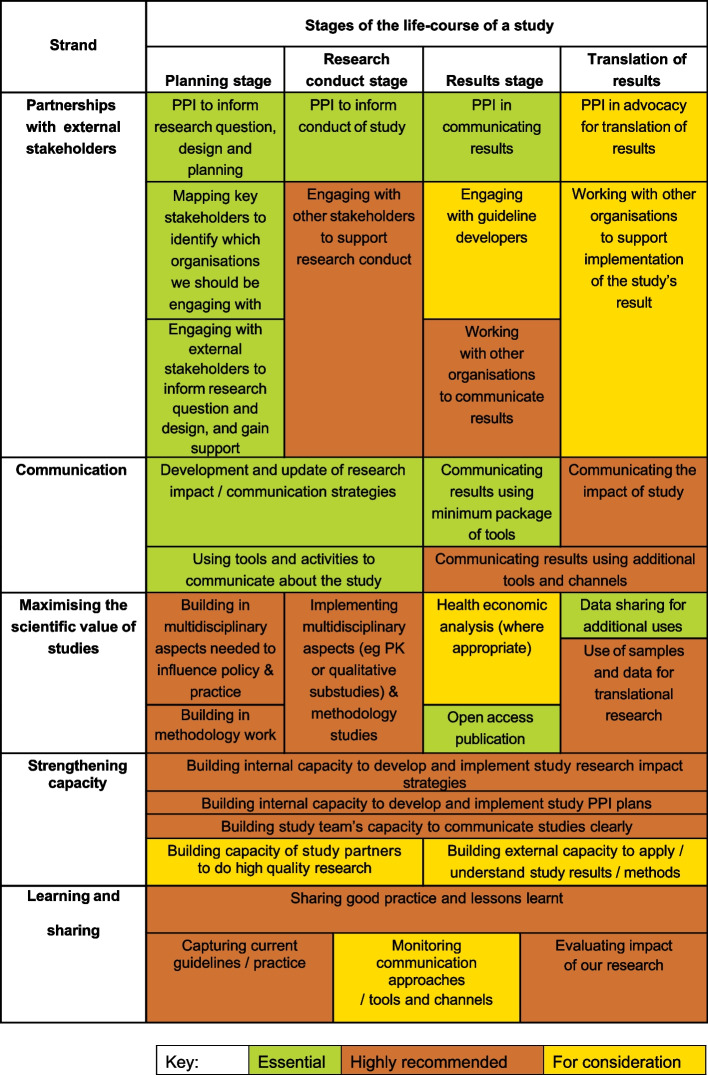
Clinical study-level knowledge transfer and exchange activities

Through discussion, the working group developed separate tables showing the activities happening at unit (Table 2) and study level (Fig. 1), organised by strand as identified earlier in the process. The Working Group then developed checklists for studies at different stages (planning (from initial idea through to opening of the study), conduct (from opening to closing of the study), results (from analysis of results to publication), and translation of results (activities that take place after publication)). The checklists contain links to relevant guidance, to help teams think through what they should be doing to encourage knowledge transfer and exchange. Examples of the different activities being applied in different studies were compiled.

**Table 2:** Unit-level knowledge transfer and exchange activities

Strand	Activities
**Partnerships with external stakeholders**	Patient and Public Involvement (PPI) Group
	PPI input to Quality Management Advisory Group
	PPI on Protocol Review Committee
	Engaging with other external stakeholders (long-term relationships lasting over generations of trials, and new partnerships developed to respond to current challenges and opportunities), including NGOs, professional bodies, guideline developers, healthcare commissioners, ethics committees, regulators and industry partners
**Communication**	Development and implementation of Unit Communications Strategy
	Maintaining communications channels including Vimeo, Soundcloud, MRCCTU website, LinkedIn, YouTube and Twitter
**Maximising the scientific value of studies**	Unit infrastructure supporting open access publication
	Unit infrastructure supporting data sharing
	SSG review to look for opportunities to embed methodology studies, and other ways to maximise the scientific value of our studies
	Identifying IP issues that need to be considered for a study
**Strengthening capacity**	Building internal capacity to develop and implement research impact strategies
	Building internal capacity to involve patients and the public in research and communication of results
	Building internal capacity to communicate research clearly
	Building external capacity to do high-quality research and apply methods developed at the unit
	Building external capacity to use/understand research
**Learning and sharing**	Seconding people into the unit with very specific skill sets to bring to the CTU, and those seeking to gain skills and experience to further their own careers within partner organisations
	Seconding unit staff to partner organisations
	Evaluating the impact of our research, and sharing case studies internally and externally
	Collect examples of impact of our research annually
	Monitoring our unit communication channels
	Sharing good practice and lessons learnt

The Knowledge Transfer and Exchange Working Group recruited studies at different stages of the trial life-cycle, to pilot the strategy, guidance and tools. Feedback led us to clarify the wording in some places, and compile examples from previous studies to illustrate some of the activities. Trial teams who piloted the worksheets found them easy to use and thought-provoking. Teams who piloted the strategy agreed with our categorisation of activities. No additional activities to include were identified through the piloting. The strategy was revised and then launched to the unit. Study teams were offered support from the Communications Team to complete the worksheets. Further feedback and examples to use in the guidance were encouraged.

## The knowledge transfer and exchange strategy

The strategy is based around five strands of activity that apply at the unit and study levels, across the life-course of our research, described in Table 1. Table 2 shows the unit-level activities under each of these strands. Figure 1 outlines the different activities that may be appropriate for our clinical studies in each of the five strands of our strategy, across the life-course of the study. Those in green are considered essential, while those in orange are highly recommended. Those in yellow are for consideration, as they might not be appropriate for every study. Supplementary Materials contains the worksheets for the different study stages.

The strategy has been incorporated into the training we provide for our staff on ‘Planning for Impact’, and has been promoted via internal meetings, on the intranet, and in the internal newsletter.

## Discussion

We developed a knowledge transfer and exchange strategy for our clinical studies, focusing on five areas of activity, across the lifecycle of a study, from planning through to translation of results:

1. Partnerships with external stakeholders (including patient and public involvement)
2. Communication
3. Maximising the scientific value of our studies
4. Strengthening capacity
5. Learning and sharing

The strategy and associated tools and guidance provide a structured approach to help study teams think through knowledge transfer and exchange at different stages of their project and record that thinking, which may be helpful when evaluating activities or reporting to funders. However, the process of completing the worksheets and implementing the activities does take time, which may be a barrier to some busy trial teams engaging with the strategy.

There are numerous models and frameworks for knowledge transfer in the published literature [6, 8–17]. Ward et al. found 28 different models in their 2009 review [7], from which they identified five common components of the knowledge transfer process, which overlap with the four research stages of our strategy (they go further than our research strategy, to research utilisation, which is beyond the scope of our strategy, as that is carried out by health care practitioners rather than researchers). Their problem identification and communication component links to some of our activities in the ‘planning stage’, particularly patient and public involvement to inform the research question; engaging with external stakeholders to inform research question and design; and building in multidisciplinary aspects needed to influence policy and practice. Their analysis of the context component is demonstrated in our activities of mapping key stakeholders to identify which organisations we should be engaging with; development of research impact strategies and capturing current guidelines/practice. Their knowledge transfer activities or interventions component could include many of the activities under the communication (‘distribution’) and partnership (‘linkage’) strands of our strategy, primarily at the results and translation of results stages.

Where our strategy differs from many of the existing knowledge transfer models is its direct application to clinical trial, observational studies and meta-analysis research, explicitly focusing on the practical actions study teams and clinical trials units can undertake throughout the research lifecourse to enable impact. Many of the existing models and frameworks focus instead on the perspective of the (potential) information user, when seeking to apply evidence in practice [9, 13, 14, 17], or identify factors for researchers to consider [8, 10, 11], or focus more narrowly on one strand of activities from our strategy [15, 16]. Our strategy considers not just the clinical implementation of study results, but also impact on science through data and sample sharing and methodological developments generated from the research. Another difference from most existing frameworks is our strategy identifies patient and public involvement as an essential part of knowledge transfer and exchange (within the partnership strand of activities), from identifying research questions through to advocating for the translation of results. As such, we hope our strategy will be of use to other researchers thinking about what they can do to maximise and speed the impact of their research.

## Conclusion

Our strategy, focusing on five strands of knowledge transfer and exchange activities across the lifecycle of clinical trials and meta-analyses, may help researchers systematically identify things they can do which may help to improve the usefulness and uptake of their study results.

## Supplementary Information

Supplementary Material 1.

## Data Availability

Worksheets generated as part of this work are available as supplementary material. No data were collected for this work.
