# Supplementary material for: Developing a knowledge transfer and exchange strategy for a clinical trials unit
Source: Trials. 2025 Jan 3;26:3. doi: 10.1186/s13063-024-08681-x (PMC11697623; doi:10.1186/s13063-024-08681-x)
Supplement: Supplementary file 1 — Supplementary Material 1. [file 13063_2024_8681_MOESM1_ESM.docx]

# Supplementary Materials 1: Worksheets

# **Worksheet 1: KTE at the planning stage (clinical studies)**

|  | **Activity** |  | **Notes / details** | **Guidance** |
| --- | --- | --- | --- | --- |
| 1.1 | Have you carried out / do you plan to carry out PPI to inform the research question, design and planning of your study? |  |  | See MRC CTU SOP 084: Patient and Public Involvement |
| 1.2 | Have you mapped the key stakeholder for your study to identify which organisations you should be engaging with? |  |  | Planning for Impact workshop 1 provides guidance on this process. |
| 1.3 | Have you engaged with external stakeholders to inform your research question and design? And/or to gain their buy-in or support for the study? (Please give details) |  |  | eg National or international policymakers, industry, professional associations, regulators & ethics committees |
| 1.4 | Have you developed a research impact / communication strategy? |  |  | Planning for Impact workshops 1 and 2 provides guidance on this process. |
| 1.5 | Have you developed communication tools / carried out activities to communicate about the study? (Please give details) |  |  | eg briefing for key stakeholders |
| 1.6 | Does your study include multidisciplinary aspects to help influence policy/practice? (If yes, please give details) |  |  | eg Translational research, health economics, pharmacokinetics, qualitative substudies |
| 1.7 | Does your study include embedded methodology work? |  |  | eg methodology studies within a trial |
| 1.8 | Have you shared any good practice/lessons learnt internally or externally? (If yes, please give details) |  |  | eg at Theme meetings / functional group meetings / conferences |
| 1.9 | Have you created a record of current guidelines / practice relating to your research question? |  |  | Planning for Impact workshop 1 provides guidance on this process. |
| 1.10 | Have members of your team undertaken training in developing/implementing study research impact strategies? |  |  | eg Planning for Impact workshops 1 and/or 2 |
| 1.11 | Have members of your team undertaken training / received support from the PPI coordinator / used tools and templates on SOPBox to develop and implement the study PPI plan? |  |  | Tools and templates associated with MRC CTU SOP 084: Patient and Public Involvement |
| 1.12 | Have members of your team received training on communication? |  |  | eg writing in plain English |
| 1.13 | As part of your study, will you be doing activities to build the capacity of study partners to do high quality research? (If yes, please give details) |  |  |  |

# **Worksheet 2:** **KTE at the research conduct stage (clinical studies)**

|  | **Activity** |  | **Notes / details** | **Guidance** |
| --- | --- | --- | --- | --- |
| 2.1 | Are you carrying out PPI activities to inform the conduct of your study? |  |  | See MRC CTU SOP 084: Patient and Public Involvement |
| 2.2 | Are you engaging with other stakeholders? |  |  | eg policymakers, professional associations, healthcare commissioners, industry |
| 2.3 | Do you have an up-to-date research impact or communication strategy? |  |  | Planning for Impact workshops 1 and 2 provides guidance on this process.  Research Impact Strategy template available as a tool associated with the Trial Reporting and Communication SOP on SOPBox |
| 2.4 | Have you developed tools / carried out activities to communicate about the study? (Please give details) |  |  | eg websites/webpages, videos, posters, information sheets, events |
| 2.5 | Does your study include multidisciplinary aspects to help influence policy/practice? (If yes, please give details) |  |  | eg Translational research, health economics, pharmacokinetics, qualitative substudies |
| 2.6 | Have you shared any good practice/lessons learnt internally or externally? (If yes, please give details) |  |  | eg at Theme meetings / functional group meetings / conferences |
| 2.7 | Have you monitored and/or evaluated any of the communications tools/channels/activities that you have used? (If yes, please give details) |  |  |  |
| 2.8 | Have members of your team undertaken training in developing/implementing study research impact strategies? |  |  | eg Planning for Impact workshops 1 and/or 2 |
| 2.9 | Have members of your team undertaken training / received support from the PPI coordinator / used tools and templates on SOPBox to develop and implement the study PPI plan? |  |  | Tools and templates associated with MRC CTU SOP 084: Patient and Public Involvement |
| 2.10 | Have members of your team received training on communication? (If yes, please give details) |  |  | eg writing in plain English |
| 2.11 | Are you doing any activities to build the capacity of study partners to do high quality research? (If yes, please give details) |  |  |  |

# **Worksheet 3:** **KTE at the results stage (clinical studies)**

|  | **Activity** |  | **Notes / details** | **Guidance** |
| --- | --- | --- | --- | --- |
| 3.1 | Are you carrying out PPI activities to inform the communication of your study? |  |  | See MRC CTU SOP 084: Patient and Public Involvement |
| 3.2 | Do you plan to engage with guideline developers? |  |  |  |
| 3.3 | Are you working with other organisations to communicate your results (If yes, please give details) |  |  | eg patient groups, professional associations, other partners |
| 3.4 | Are you using at least the minimum package of tools to communicate your results? |  |  | Details of the minimum package are available on the intranet |
| 3.5 | Are you using additional tools and channels to communicate your results (if yes, please give details) |  |  | eg press release, videos, podcasts, infographics, events |
| 3.6 | Is health economics being carried out linked to your study? |  |  | This may not be appropriate, depending on what your results showed |
| 3.7 | Do your peer reviewed publications comply with the UKRI Open Access requirements? |  |  | Guidance available on the intranet |
| 3.8 | Have you shared any good practice/lessons learnt internally or externally? (If yes, please give details) |  |  | eg at Theme meetings / functional group meetings / conferences |
| 3.9 | Have you monitored and/or evaluated any of the communications tools/channels/activities that you have used? (If yes, please give details) |  |  |  |
| 3.10 | Have members of your team undertaken training in developing/implementing study research impact strategies? |  |  | eg Planning for Impact workshops 1 and/or 2 |
| 3.11 | Have members of your team undertaken training / received support from the PPI coordinator / used tools and templates on SOPBox to develop and implement the study PPI plan? |  |  | Tools and templates associated with MRC CTU SOP 084: Patient and Public Involvement |
| 3.12 | Have members of your team received training on communication? (Eg. writing in plain English) (If yes, please give details) |  |  | eg writing in plain English |
| 3.13 | Are you doing any activities to build the capacity of external stakeholders to apply or understand your study results or methods? (If yes, please give details) |  |  |  |

# **Worksheet 4:** **KTE at the translation of results stage (clinical studies)**

|  | **Activity** |  | **Notes / details** | **Guidance** |
| --- | --- | --- | --- | --- |
| 4.1 | Are you carrying out PPI activities around the translation of results into policy and practice? (If yes, please give details) |  |  | See MRC CTU SOP 084: Patient and Public Involvement |
| 4.2 | Are you working with other organisations to support implementation of the study’s results? (if yes, please give details) |  |  | eg patient groups, professional associations, other partners |
| 4.3 | Have you done any work to communicate the impact of your study? (If yes, please give details) |  |  | eg REF case study, or case study for QQR report |
| 4.4 | Are you using additional tools and channels to communicate your results (if yes, please give details) |  |  | eg press release, videos, podcasts, infographics, events |
| 4.5 | Do your study publications (and, if relevant, website) explain how people can request data sharing? |  |  | See MRC_CTU_SOP_061 Data Sharing and Data Reuse (available on SOPBox) and <https://www.mrcctu.ucl.ac.uk/our-research/other-research-policy/data-sharing/> |
| 4.5b | Have you had requests for sharing of data? (If yes, please give details) |  |  |  |
| 4.6 | Have your samples and data been used for translational research? (If yes, please give details) |  |  |  |
| 4.7 | Have you shared any good practice/lessons learnt internally or externally? (If yes, please give details) |  |  | eg at Theme meetings / functional group meetings / conferences |
| 4.8 | Have you evaluated the impact of the study? (If yes, please give details) |  |  | Planning for Impact workshop 3 covers this. |
| 4.9 | Have members of your team undertaken training in developing/implementing study research impact strategies? |  |  | eg Planning for Impact workshops 1 and/or 2 |
| 4.10 | Have members of your team undertaken training / received support from the PPI coordinator / used tools and templates on SOPBox to develop and implement the study PPI plan? |  |  | Tools and templates associated with MRC CTU SOP 084: Patient and Public Involvement |
| 4.11 | Have members of your team received training on communication? (If yes, please give details) |  |  | eg writing in plain English |
| 4.12 | Are you doing any activities to build the capacity of external stakeholders to apply or understand your study results or methods? (If yes, please give details) |  |  |  |
